# 癌相关成纤维细胞来源的外泌体在肺恶性肿瘤中的研究进展

**DOI:** 10.3779/j.issn.1009-3419.2021.102.07

**Published:** 2021-03-20

**Authors:** 诚 沈, 珏 李, 佳龙 李, 国卫 车

**Affiliations:** 610041 成都，四川大学华西医院胸外科 Department of Thoracic Surgery, West China Hospital, Sichuan University, Chengdu 610041, China

**Keywords:** 肿瘤微环境, 癌相关成纤维细胞, 外泌体, 肺肿瘤, Tumor microenvironment, Cancer-associated fibroblasts, Exosomes, Lung neoplasms

## Abstract

肿瘤微环境（tumor microenvironment, TME）是分布在肿瘤细胞周围的动态网络，癌相关成纤维细胞（cancer-associated fibroblasts, CAFs）作为肿瘤微环境中的一部分，不但与正常成纤维细胞密切相关，而且可以分泌多种物质（包括外泌体）参与肿瘤微环境的调控。外泌体不仅与肺恶性肿瘤微环境形成密切相关，同时其可增加肺癌细胞侵袭与转移能力，对肿瘤免疫抑制有介导作用，还参与肺癌患者接受放化疗后耐药的机制中。本文就CAFs来源相关外泌体在肺癌中的研究现状和进展做一综述。

## Abstract (en)

Tumor microenvironment (TME) is a dynamic network distributed around tumor cells. Cancer-associated fibroblasts (CAFs), as an important component of the TME, are not only closely related to normal fibroblasts, but also can secrete a variety of substances to participate in the regulation of the TME. Exosomes, one of the substances from CAFs, can promote the formation and development of lung cancer, including promoting the formation of TME, increasing pulmonary tumor cell invasion and metastasis, mediating pulmonary tumor immunosuppression and participating in radiotherapy and chemotherapy resistance. This article reviews the current research status and progress of cancer associated fibroblasts-derived exosomes in lung cancer.

肿瘤微环境（tumor microenvironment, TME）是分布在肿瘤细胞周围的动态网络，是指肿瘤基质中的各种细胞包括成纤维细胞、免疫细胞、间充质细胞以及附近区域的间质和浸润在其中的生物分子等组成的胞外环境^[1, 2]^。肿瘤细胞是“种子”，其周围的细胞及非细胞组份构成“土壤（肿瘤微环境）”，因“土壤”和“种子”特殊的内在关系，“肿瘤微环境”现已成为肿瘤精准治疗研究的关键靶点^[3]^。

近年来，国内外相关专家学者研究发现，在组织创伤与炎症修复过程中同样存在着成纤维细胞表型转换^[4]^。通常情况下研究者认为，癌细胞分泌的因子为了营造肿瘤微环境，即为肿瘤细胞生长营造一个合适的微环境，常常促进成纤维细胞表型转换的发生^[5]^。作为构成肿瘤微环境的关键要素，癌相关成纤维细胞（cancer-associated fibroblasts, CAFs）不但与正常成纤维细胞密切相关，而且可以分泌多种物质参与肿瘤微环境的调控^[6]^。近年来研究^[7]^提示，CAFs分泌的物质中就包括外泌体，该种物质一方面能够通过营造适合肿瘤生长的微环境而使癌细胞具有更强的转移与侵袭能力，另一方面还能够介导肿瘤免疫抑制，从而在一定程度上推动肿瘤的发生发展。本文就CAFs来源相关外泌体在肺恶性肿瘤中的研究现状和进展做一综述。

## 1 CAFs概述

### 1.1 CAFs的来源

关于CAFs的来源^[8-10]^，就现阶段已发表的研究成果来看，主要有以下几种来源：①在癌细胞分泌的血小板衍生因子、转化生长因子β（transforming growth factor β, TGF-β）等各种细胞因子作用下，由常驻成纤维细胞，即剩余正常间质中的成纤维细胞诱导分化形成; ②骨髓间充质干细胞。它被视为CAF前体细胞的一种; ③血管外膜细胞与血管平滑肌细胞; ④上皮-间质转化（epithelial-mesenchymal transition, EMT），如TGF-β1能够诱导内皮细胞发生表型转化为成纤维细胞; ⑤脂肪细胞。据有关学者研究发现，肿瘤细胞中存在着大量脂肪细胞，其中脂肪干细胞直接与肿瘤细胞相互作用从而促进成纤维细胞的分化。总之，通过对不同CAFs起源的认识，可以促进和帮忙我们了解不同的CAFs的表型和功能。

### 1.2 CAFs异质性

据研究成果表明，细胞表型的差异是CAFs异质性的主要体现，具体来讲，CAFs表型转换具有两种特性：其一为时相性，主要反映在肿瘤发展阶段与表型转换密切相关; 其二为空间特性，一方面是指癌组织不同部位的成纤维细胞具有不同的表型，另一方面是同一成纤维细胞在组织内各种部位的差异化表型体现^[11]^。同时，随着相关技术的发展，在单细胞水平上定量分析细胞转录组的差异成为可能。研究者利用单细胞RNA测序技术，在小鼠乳腺癌模型中，按照CAFs亚群的基因可将其分为以下四群^[12]^：①mCAF亚群，其由常驻组织成纤维细胞转换而来; ②dCAF亚群，其来源于肿瘤上皮间质; ③vCAF亚群，其由血管周细胞转换而来; ④cCAF亚群，其与vCAF重叠，与此同时还具有较强的增殖能力。

### 1.3 CAFs的功能及作用

CAFs可以通过建立并重塑细胞外基质结构，使肿瘤细胞易于侵入血管^[13]^; 通过分泌多种生长因子、细胞因子和趋化因子，CAFs可与肿瘤细胞或其他基质细胞相互作用，促进肿瘤的进展^[14]^。同时，CAFs也被认为是开发新型抗癌药物的重要靶标^[15]^。其主要作用包括：①CAFs可以促进肿瘤的发生; ②CAFs促进肿瘤的侵袭和转移; ③CAFs可以促进肿瘤血管的生成; ④CAFs促进肿瘤的耐药。

## 2 外泌体概述

作为一种细胞外细胞器，细胞外囊泡主要来源于真核细胞，首次提及则是在1983年Gurunathan等^[16]^研究网织红细胞成熟的过程中发现了一种和转铁蛋白受体相关的膜型囊泡（vesicles）被分泌到细胞外，并于4年后在超速离心下获得。其主要包括外泌体（exosomes）、脱落微泡（shed microvesicle, SMV）和凋亡小体（apoptosis body, AB）三种，而外泌体是其中最为重要的一种^[17]^。有文献^[18]^指出，外泌体一方面能够通过营造适合肺癌发生发展的微环境而使癌细胞具有更强的转移与侵袭能力，另一方面还能够介导肿瘤免疫抑制，从而在一定程度上推动肿瘤的发生发展。除此之外，外泌体在恶性肿瘤微环境中，与肥大细胞、恶性细胞等互相交互摄取并将外泌体携带的诸多成分转移至细胞质，还可靶向调节细胞中mRNA、蛋白等水平，改变细胞的生物学特性^[19]^。

### 2.1 外泌体的提取与分离

外泌体分离方法一般是基于其物理化学特性，包括大小、密度、质量、表面蛋白等特点。常见的方法包括了超速离心法、蔗糖密度梯度离心法、商品化试剂盒沉淀法、聚合物沉淀法、免疫磁珠法和微流控芯片法^[20, 21]^。

超速离心法分离外泌体是目前最普遍的一种方法，但在分离过程中，由于超高速离心，外泌体的结构、功能很容易遭到破坏，对结果分析产生不利; 蔗糖密度梯度离心法是超速离心法的改进与优化，但依然存在和超速离心法相类似的结果^[17, 22]^。商品化试剂盒沉淀法不仅操作简便，同时也不需要特殊的设备，然而市面上目前有的商品化试剂盒价格较为昂贵，不利于大批量提取外泌体^[17, 22]^。聚合物沉淀法操作过程虽然不涉及大型昂贵仪器设备的协助，但易受到共沉淀的其他物质的污染，影响下游分析^[23, 24]^。免疫磁珠法分选具有特异性强、纯度较高、不影响外泌体结构形态的特点，但是其仍存在效率低，磁珠费用高昂的缺点，难以普及^[23]^。近年来利用微流控芯片来分离外泌体得到众多学者的关注^[25]^。一方面，微流控技术能够分离小体积样品中的外泌体，有助于临床前肿瘤小动物模型试验的研究; 另一方面，微流体技术自动化程度高，所需样品量小，相较于超速离心法更适合临床应用。

### 2.2 外泌体的生物学特点

在经过一系列调控过程如“内吞-融合-外排”等后，细胞能够形成30 nm-150 nm大小的囊泡，此即为外泌体，无论是在正常的生理条件下，还是在病理条件下，外泌体都能产生，在电子显微镜下外泌体呈现典型的50 nm-100 nm杯状或蝶形脂质双分子膜结构^[26]^。相关研究^[27, 28]^结果表明，外泌体具有热休克蛋白70、凋亡相关基因-2（apoptosis-linked gene-2, ALG-2）相互作用蛋白X、四次跨膜蛋白（CD9、CD63、CD81）、肿瘤易感基因101蛋白等特定的表面分子特征，同时其存在于几乎所有的人体液体中。

### 2.3 外泌体的生物学功能

外泌体含有多种蛋白质、核酸、甚至病毒等遗传物质，这些内含物可以通过外泌体转移到受体细胞，能调节受体细胞的蛋白质表达和信号通路的改变，在细胞间物质和信息转导中起重要作用^[29, 30]^，主要包括：①外泌体能作为信号复合物通过受体介导对靶细胞产生直接的刺激作用; ②可将多种活性生物成分如细胞器、蛋白质等转运至靶细胞内，从而实现受体细胞转换过程以及发挥其改变细胞功能状态的作用，这一系列过程是通过影响靶细胞基因转录、翻译，调节其信号通路而实现的。此外，外泌体还具有免疫调节等功能。

## 3 CAFs与肺癌的相关性

CAFs可以通过建立并重塑细胞外基质结构，促使肿瘤血管的生成。研究^[31]^发现CAFs与新生血管相关的基质细胞衍生因子1（stromal derived factor 1, SDF-1）有密切相关性，其可以募集内皮祖细胞来促进血管的生成。在肿瘤转移方面，CAFs也起到至关重要的角色。CAFs中高表达的白细胞介素6可以激活肺癌细胞Janus激酶2/信号转导与转录激活子3信号通路，促进肺癌细胞上皮间质转化过程，上调转移相关基因如基质金属蛋白酶2的表达，从而明显地增强肺癌细胞的转移潜能和活性^[32]^。此外，CAFs也促进肺癌的耐药和免疫。CAFs促进肺癌细胞系A549产生胰岛素样生长因子-2来介导耐药^[15]^。另一项研究发现，白细胞介素-11在顺铂治疗后的肺腺癌患者癌灶内受到上调的作用，进一步激活抗凋亡途径及肺癌细胞耐药性^[33]^。结果还表明，白细胞介素-11高表达的患者对顺铂的反应较低表达的患者差。

## 4 外泌体与肺癌的相关性

外泌体在肺癌微环境形成的方面有促进作用，同时还能增强肿瘤细胞的侵袭能力^[34]^。伴随着癌基因的不稳定性，低氧、酸中毒等因素的影响以及炎症免疫反应等诱因均能促进肿瘤细胞释放外泌体形成肿瘤微环境。此外，肿瘤细胞来源的外泌体可以调控其自身的免疫状态。参与的主要机制是通过携带免疫抑制因子并抑制免疫细胞发挥的抗肿瘤免疫效应，诱导免疫抑制和调节细胞群，例如调节性T细胞和调节性B细胞等^[35, 36]^。此外，外泌体也参与免疫逃逸作用，主要是通过转导特定蛋白和RNA进入受体细胞来进行发挥^[37]^。因此，外泌体可通过多途径参与肺癌的发生和发展。

在肺癌诊断中外泌体RNA和蛋白质也有一定作用。肺癌患者中，外泌体RNA水平明显高于健康者，且RNA水平与肺癌的进展程度、生存率都有密切关系^[38]^。研究发现血液循环中肺癌来源的外泌体相关的miRNA等对诊断有较高价值^[38, 39]^。肺癌细胞来源的外泌体中富集多种蛋白质成分，在肺癌发生发展方面有明显促进作用，可能是早期诊断肺癌的有效方法和途径。

外泌体在肺癌治疗中也有一定意义。目前肺癌的治疗包括了手术、化学治疗、放射治疗、分子靶向治疗，但针对不同时期的肺癌患者所采用的各种治疗方式均存在局限性，同时肺癌患者存在早期复发和转移可能的特点，导致患者预后往往较差。研究发现，基于外泌体的研究可为肺癌患者的治疗带来新希望。外泌体不仅在化疗药物的敏感性方面有增强作用，同时还可以抑制癌基因的表达，促进肺癌细胞的凋亡，因此其成分和功能也为肺癌的治疗提供多个潜在治疗靶点。研究^[40]^发现在肺腺癌中，癌细胞来源的外泌体所包含的miR-181a能增加肺腺癌细胞对紫杉醇和铂类化疗的敏感性。通过使用酶抑制剂和去甲基化药物可降低癌细胞来源的外泌体所包含的miR-512和miR-373活性，继而增加肺癌细胞对铂类的化疗敏感性^[41]^。

## 5 CAFs来源的外泌体在肺癌中的作用

肺癌作为一种高度致死性的恶性肿瘤，占所有新增癌症病例的13.2%，占所有癌症相关死亡的25.9%。外泌体的组成较为复杂，通过进入血液循环，而后被靶细胞吸收，从而调节靶细胞基因表达和细胞功能。此外，外泌体相关的miRNA作为短单链和非编码RNA分子，调节致癌基因或抑癌基因的表达，参与细胞分化、细胞凋亡及细胞信号的传导。

肺癌细胞来源的外泌体在肺癌发生发展过程中的报道较多^[7, 42-44]^，而有关肺癌CAFs来源的外泌体研究报道较少。You等^[45]^利用对照培养基的方式，采用qRT-PCR和Western blot检测手段对肺癌细胞中上皮间质转化相关蛋白水平进行检测，结果发现CAFs可通过释放的外泌体来促进肺癌细胞中的上皮间质转化（epithelial-mesenchymal transition, EMT）。类似的结果在Yang等^[46]^研究中有报道，CAFs来源的外泌体包含有高表达的miR-210，同时其通过靶向UPF1来激活PTEN/PI3K/AKT通路以促进EMT，从而促进非小细胞肺癌的迁移、增殖和侵袭能力。同时，CAFs分泌的外泌体中SNAI1水平与CAFs中的SNAI1表达有相关性。此外，外泌体中SNAI1的水平对于诱导肺癌细胞中的EMT起重要促进作用。用外泌体释放抑制剂GW4869处理CAFs后可明显抑制它们对受体上皮细胞的EMT诱导作用。Guo等^[47]^研究发现肺鳞癌CAFs来源的外泌体中miR-369对肺鳞癌细胞迁移，侵袭和肿瘤发生有相关性，miR-369可能是肺鳞癌重要的预后标志物和治疗靶标。

## 6 展望

在肿瘤微环境中，CAFs是肺癌基质内的重要细胞成分，外泌体是多种细胞间的沟通工具，CAFs和肿瘤来源的外泌体研究较为广泛。但是，CAFs来源的外泌体在恶性肿瘤中的作用机制依然缺乏深入研究，尤其是在肺癌的相关研究中相对空缺。因此，有关CAFs来源的外泌体研究有着广阔的前景，深入研究肺癌微环境、CAFs和与之相关来源的外泌体三者间的关系，将更有利于了解肺部恶性肿瘤的恶性进程、耐药等作用机制，从而造福更多的患者。
